# Nanobiotechnology in combating CoVid-19

**DOI:** 10.6026/97320630016828

**Published:** 2020-11-30

**Authors:** Dhanraj Ganapathy, Rajeshkumar Shanmugam, Lakshmi Thangavelu

**Affiliations:** 1 Department of Prosthodontics, Saveetha Dental College, Saveetha Institute of Medical and Technical Sciences, Chennai - 600 077, India; 2 Department of Pharmacology, Saveetha Dental College, Saveetha Institute of Medical and Technical Sciences, Chennai - 600 077, India;

**Keywords:** Nanotechnology, carbon dots, CoVid-19, disease, virus

## Abstract

Emergence of novel pandemic viral disease CoVid-19 and its mutational behaviour are alarming. The potential use of nano-biotechnology in combating CoVid-19 is promising. We glean available data to explore such possibility in this short note.

## Nanobiotechnology in combating CoVid-19:

Hybrid nanosystems are structured amalgamations of inorganic - organic, inorganic - inorganic (nanocomposites), and organic - organic nanoparticulated systems (lipid - polymer hybrid NPs) designed on the basis of particular requirements or usage at the targeted site [1]. Huo C et al. have reported zirconia nano particles offered protection against pathogenic avian influenza virus and suggested the possibility of using it against a variety of infectious conditions [2]. Kumar et al. (2019) developed iron oxide nanoparticles (IO-NPs) with particle size ranging from 10-15 nm against the pandemic influenza virus strain A/H1N1/Eastern India/66/PR8-H1N1. The antiviral effectiveness was estimated using RT-PCR. Considerable reductions in viral concentration were observed when treated with Iron oxide nanoparticles [3]. This has potential application in the management of CoVid-19. Hu et al. (2018) reported that nanoparticle encapsulation of diphyllin and bafilomycin improved anti-influenza activity in animal models demonstrating the therapeutical potential of the nanoparticulate V-ATPase inhibitors for host-targeted treatment against influenza [4]. Haggag et al.(2019) used the aqueous and hexane extracts of Lampranthus coccineus and Malephora lutea F. Aizoaceae for the synthesis of silver nanoparticles [5]. They showed that silver nano particles of L.coccineus and M. lutea have antiviral activity against HSV-1, HAV-10, and CoxB4 virus. Wang et al. (2019) developed aptamer-antibody on a multiwalled carbon nanotube-gold conjugated sensing surface with a dielectrode to detect pandemic H1N1 [6]. The application of nanomedicine in the design and development of vaccine candidates and formulation is promising [7]. Ding et al. (2019) documented Cap-3M2e VLP as a bivalent nanovaccine with dual IAV and PCV2 protection [8]. Pons-Faudoa et al. (2019) formulated a subcutaneously embedded nanofluidic component for the sustained release of antiviral drug, cabotegravir , an integrase inibitor (CAB) conjugated with 2-hydroxypropyl-β-cyclodextrin (βCAB) [9]. They observed βCAB treatment inhibited viral replication by 90% and demonstrated the potential of continuous release of βCAB via a nanofluidic implant for viral infections including COVID 19 [9]. Lcczechin et al. (2019) used seven various carbon quantum dots (CQDs) to estimate antiviral efficacy in human coronavirus HCoV-229E infections [10]. Chowdhury et al. (2019) developed a pulse-triggered ultrasensitive electrochemical sensor utilizing graphene quantum dots with gold-embedded polyaniline nanowires for the diagnosis of hepatitis E virus [11]. Huang et al. (2019) formulated benzoxazine monomer extracted carbon dots (BZM-CDs) and demonstrated their successful anti-viral exercise against Japanese encephalitis, ZIKA, dengue and non-enveloped viruses including swine parvovirus and adenoviruses [12]. Qaddare et al. (2017) introduced a new carbon dot-conjugated nanosensor with high biocompatibility and non-toxicity for the identification of other DNA biomarkers [13]. Lee et al. (2015) established plasmon-assisted fluoro-immunoassay (PAFI) for the detection of the flu virus uisng carbon nanotube adorned Au nanoparticle (Au NP) [14]. Lou (2015) developed a strategy for the detection of DNA by highly sensitive electro chemi luminescence (ECL) based on the site-specific cleavage of BamHI endonuclease combined with the ECL of graphene quantum dots (GQDs) and bidentate chelation of dithiocarbamate DNA (DTC-DNA) test assembly [15]. Thus, carbon quantum dots are a potential tool in the diagnosis and treatment of CoVid-19.

## Conclusion:

Nanobiotechnology has enormous promise in the management of CoVid-19. Nanotechnology coupled with specific biomarkers has potentoial application in the clinical diagnosis and treatment of CoVid-19.
